# Stellate Ganglion Block and Intraarterial Spasmolysis in Patients with Cerebral Vasospasm: A Retrospective Cohort Study

**DOI:** 10.1007/s12028-023-01762-w

**Published:** 2023-07-27

**Authors:** Christopher Wendel, Cornelia Oberhauser, Jan Schiff, Hans Henkes, Oliver Ganslandt

**Affiliations:** 1 Neurosurgical Clinic, Klinikum Stuttgart, Kriegsbergstr. 60, 70174 Stuttgart, Germany; 2 Institute for Medical Information Processing, Biometry, and Epidemiology, Chair of Public Health and Health Services Research, Ludwig-Maximilians-University, Munich, Germany; 3 Pettenkofer School of Public Health, Munich, Germany; 4 Department of Anesthesiology and Operative Intensive Care Medicine, Klinikum Stuttgart, Stuttgart, Germany; 5 Neuroradiological Clinic, Klinikum Stuttgart, Stuttgart, Germany; 6 Department of Neurosurgery, Friedrich-Alexander-Universität Erlangen-Nürnberg, Schwabachanlage 6, 91054 Erlangen, Germany

**Keywords:** Cerebral vasospasm, Delayed cerebral ischemia, Delayed ischemia, Stellate ganglion block, SGB, Intraarterial spasmolysis, Chemical angioplasty, Subarachnoid hemorrhage

## Abstract

**Background:**

In patients with symptomatic cerebral vasospasm (CV) following aneurysmal subarachnoid hemorrhage who do not respond to medical therapy, urgent treatment escalation has been suggested to be beneficial for brain tissue at risk. In our routine clinical care setting, we implemented stellate ganglion block (SGB) as a rescue therapy with subsequent escalation to intraarterial spasmolysis (IAS) with milrinone for refractory CV.

**Methods:**

In this retrospective analysis from 2012 to 2021, patients with CV following aneurysmal subarachnoid hemorrhage who received an SGB or IAS were identified. Patients were assessed through neurological examination and transcranial Doppler. Rescue therapy was performed in patients with mean cerebral blood flow velocity (CBFV) ≥ 120 cm/s and persistent neurological deterioration/intubation under induced hypertension. Patients were reassessed after therapy and the following day. The Glasgow Outcome Scale was assessed at discharge and 6-month follow-up.

**Results:**

A total of 82 patients (mean age 50.16 years) with 184 areas treated with SGB and/or IAS met the inclusion criteria; 109 nonaffected areas were extracted as controls. The mean CBFV decrease in the middle cerebral artery on the following day was − 30.1 (± 45.2) cm/s with SGB and − 31.5 (± 45.2) cm/s with IAS. Mixed linear regression proved the significance of the treatment categories; other fixed effects (sex, age, aneurysm treatment modality [clipping or coiling], World Federation of Neurological Surgeons score, and Fisher score) were insignificant. In logistic regression, the presence of cerebral infarction on imaging before discharge from the intensive care unit (34/82) was significantly associated with unfavorable outcomes (Glasgow Outcome Scale ≤ 3) at follow-up.

**Conclusions:**

Stellate ganglion block and IAS decreased CBFV the following 24 h in patients with CV. We suggest SGB alone for patients with mild symptomatic CV (CBFV < 180 cm/s), while subsequent escalation to IAS proved to be beneficial in patients with refractory CV and severe CBFV elevation (CBFV ≥ 180 cm/s).

**Supplementary Information:**

The online version contains supplementary material available at 10.1007/s12028-023-01762-w.

## Introduction

Delayed cerebral Ischemia (DCI) is a major thread in neurointensive care following aneurysmal subarachnoid hemorrhage (aSAH). Inadequate vascular response, leading to cerebral vessel narrowing, is thought to be one cause of DCI and could lead to cerebral infarction. Infarctions within 6 weeks of aSAH are the primary determinant of long-term outcomes and are associated with health care costs 30% higher than those in the absence of DCI [1–3].

Early detection of DCI through neurological examination is crucial, but patients with high-grade aSAH often require sedation and mechanical ventilation. In intensive care routine, transcranial Doppler (TCD) is helpful as bedside screening in these critical ill patients. Fifty-seven percent of patients with elevated cerebral blood flow velocity (CBFV) above 120 cm/s (mean) in the middle cerebral artery (MCA) measured with TCD develop DCI, compared with 30% of patients with angiographic vasospasm (50% decrease in the diameters of large vessels) [1, 4, 5].

First-line treatment of symptomatic cerebral vasospasm (CV) to prevent DCI includes induced hypertension, which significantly increases regional cerebral blood flow and brain tissue oxygenation, thus decreasing neurological deficits. Current guidelines suggest that endovascular intervention should be performed early, if disabling neurological deficits persist after maximum medical therapy [1, 6, 7].

Intraarterial spasmolysis (IAS) performed with various agents leads to a robust angiographic response. However, outcomes have not been demonstrated to be superior to those of medical management alone in patients with CV [1, 8, 9].

In recent years, stellate ganglion block (SGB) has been proposed as a readily available bedside treatment for early-stage CV. SGB is easily administered, its effectiveness in symptomatic CV has been reported in several case series, and its complication rate is low (1.7 severe complications per 1000 SGBs). The mechanism relies on blocking cervical ganglia, which supply sympathetic fibers to pial vessels, and reducing the sympathetic tonus of the cerebral vasculature leading to the reduction of ipsilateral vasoconstriction [10–16]. Recent studies have shown the importance of rapid and early treatment in patients with DCI, and a bedside treatment is potentially valuable [17, 18].

The purpose of this study was to examine lasting effects of SGB and IAS with milrinone on CBFV, in comparison with the untreated contralateral side. In addition, we analyzed available outcome metrics (Glasgow Outcome Scale, radiologic imaging) and contextualized it with available literature.

## Materials and Methods

In this retrospective cohort study, patients with aSAH and cerebral vasospasms who received an SGB or IAS between 2012 and 2021 were identified through our critical care patient data management system (COPRA PDMS, Version 6; Copra System GmbH). The local ethics committee of Baden-Wuerttemberg (Germany) approved the study (No. F-2016–107), and no informed consent was required. The reporting is in accordance with the Strengthening the Reporting of Observational Studies in Epidemiology checklist.

All patients were treated according to our aSAH protocol with oral or intravenous nimodipine (360 mg/day or 2 mg/h intravenously) for 21 days. Standard monitoring included five-lead electrocardiography, arterial oxygen saturation, continuous invasive arterial blood pressure monitoring, temperature, and fluid balance measurements. Arterial blood samples were obtained daily. End-tidal carbon dioxide levels and intracranial pressure were monitored in intubated patients. Neurological examinations were conducted four times per day. An experienced neurosurgeon performed routine TCD ultrasound examinations at approximately the same time of day.

According to the literature, the cutoff value for beginning CV was set at 120 cm/s mean blood flow velocity in the MCA or an increase of more than 40 cm/s over the course of 24 h. In awake patients, new neurological deficits were the most critical indicator of DCI. Computed tomography angiography were performed routinely if patients were intubated and DCI was suspected.

As first-line treatment, mean arterial pressure was raised to 80–100 mm Hg with vasopressor agents. Reassessment through TCD was performed on the following day, and neurological examinations were done at least four times a day in these patients.

When neurological deficits persisted despite medical treatment and the CBFV in TCD remained above 120 cm/s, an SGB was performed on the affected side. In intubated patients with elevated CBFV on the left and right sides, SGB injections were performed on both sides. SGB was performed by an intensive care specialist with 10–15 ml ropivacaine 0.2%. To extend block duration in patients with low-to-moderate vasopressor dependence, 75–150 µg clonidine was added.

If SGB lead to a reduction of CBFV in TCD after 2 h, the next TCD was performed the following day. Patients received subsequent angiography if CBFV did not respond to SGB. When vessel narrowing was observed (≥ 50% reduction of diameter), IAS was performed on the affected area in the neuroradiology department. IAS was done with 8 mg milrinone over 30 min through an infusion pump. A maximum of three areas (anterior left, anterior right, and posterior circulation) could be treated in a single session.

Patients received primary IAS if regular angiography was already scheduled and they developed CV in TCD on that day, or if they did not respond to prior SGB. If severe CV persisted after IAS, patients received continuous intraarterial nimodipine infusion through microcatheters.

If multiple episodes with CV occurred within the intensive care unit (ICU) stay, up to three IAS and five SGBs were administered.

The CBFV values of MCA on both sides before treatment, 2 h after treatment, and on the following days were recorded. Extremely low CBFV values (≤ 30 cm/s) were considered mismeasured and were excluded from analysis. In agreement with other publications, our cutoff for MCA CBFV before treatment was ≥ 120 cm/s, and lower CBFV values were excluded. If patients were treated on one side, the nonelevated and not treated contralateral side was used as a control. The difference in CBFV before treatment and on the following day (24 h) was calculated in cm/s [1].

In addition, the World Federation of Neurological Surgeons (WFNS) score and Fisher score at admission and the location of the aneurysm side (anterior or posterior circulation, left or right if applicable) were recorded. The Glasgow Outcome Scale (GOS) was recorded at discharge and at 6–12-month follow-ups. Computed tomography and magnetic resonance imaging scans of the brain obtained during the ICU stay were screened for cerebral infarction, based on the 2010 consensus definition of cerebral infarction (Supplementary Data 1), and the day of first occurrence was recorded [19].

Age at the time of the incident was calculated in years. The times from the incident to aneurysm treatment and to CV treatment and the length of stay in the ICU were calculated in days.

### Statistical analysis

Treatments were retrospectively classified into SGB, IAS, and no treatment (contralateral values). The reference category was no treatment. Patients with refractory CV could be treated multiple times, potentially with different treatments, during the ICU stay.

To explain the difference in the MCA CBFV before and after treatment, a mixed linear regression model was created, including the following fixed effects: treatment, sex, age, treatment of aneurysm (clipping/coiling), WFNS, and Fisher score, and a random intercept relating to the participating patient. Initially, the effect of frequency of treatments and random slope with treatment frequency were included in the model; however, variables did not increase the value of the model. For model fitting, restricted maximum likelihood was used. Visual inspection of residual plots did not reveal any apparent deviations from homoscedasticity or normality.

In addition, subanalysis was performed to evaluate the effect of clonidine addition to SGB on the decrease in CBFV after SGB treatment. Additionally, a subanalysis evaluating the effect of combined treatment on the decrease of CBFV in IAS patients was performed.

To reveal possible predictors of GOS at follow-up, we performed logistic regression for unfavorable GOS (≤ 3), including the following parameters: age, sex, cerebral infarction at discharge, treatment of aneurysm (clipping/coiling), WFNS, and Fisher score. The 95% confidence interval values were calculated.

Statistical calculations were conducted in R (R Core Team, 2022). The linear mixed models were run in R with the lme4 package [20]. The figures were created with the ggplot2 package [21]. Output for mixed models was created with sjPlot: Data Visualization for Statistics in Social Science [22]. Boxplots were created with the R package ggpubr: ‘ggplot2’ Based Publication Ready Plots [23].

## Results

Among 682 patients with aSAH treated in our neurovascular center from 2012 to 2021, 114 patients with CV were identified. Eighty-two patients (51 women and 31 men) with a mean age of 50.2 years (range 17.5–73.3 years) met the inclusion criteria (Supplementary Methods 2). Data for these patients (Table 1, Supplementary Table S1) comprised 184 treated areas (Table 2, Fig. 2a, b). The IAS group includes IAS only, and SGB plus subsequent IAS on the same day (22 patients with 33 areas treated). A control group comprised TCD values of unaffected areas (contralateral side; *n* = 109).

**Table 1:** Descriptive data of study population

Display	Demographic	Women	Men	All
Count	*n*	51	31	82
	Aneurysm location (AC/PC/none)	46/5/0	24/6/1	70/11/1
	Coiling/clipping/none	34/16/1	16/11/4	50/27/5
	Cerebral infarction	25	9	34
Mean (± SD)	Age (years)	49.60 (± 11.30)	51.10 (± 9.54)	50.16 (± 10.70)
	Days to aneurysm treatment	0.88 (± 1.72)	1.19 (± 1.92)	1.00 (± 1.79)
	ICU LOS (days)	23.70 (± 8.80)	24.10 (± 12.10)	23.82 (± 10.09)
Median (IQR)	WFNS scale	4 (2–5)	4 (2.5–5)	4 (2–5)
	Fisher scale	4 (3–4)	4 (4–4)	4 (3–4)
	GOS at discharge	3 (2–4)	3 (2–4)	3 (2–4)
	GOS at follow-up (*n* = 74)	4 (3–5)	4 (2.75–5)	4 (3–5)

*AC* anterior circulation, *GOS* Glasgow Outcome Scale, *IQR* Interquartile range (25th–75th percentile), *LOS* length of stay, *PC* posterior circulation, *SD* standard deviation, *WFNS* World Federation of Neurological Surgeons

**Table 2:** Means and SDs of the CBFV decrease in each group

Parameter	Count		Mean day of treatment	CBFV in cm/s			CBFV in percentage	
	Patients	Areas		Mean MCA pre	Mean MCA after 24 h	Mean MCA difference 24 h	Mean MCA decrease (%)	Decrease persistent after 24 h (%)
Stellate ganglion block	60	109	7.3	165.8 (± 29.3)	135.7 (± 39.5)	− 30.1 (± 45.2)	− 18.2	67.9
Intraarterial spasmolysis	46	75	8.3	170.2 (± 39.3)	138.6 (± 44.4)	− 31.5 (± 45.2)	− 18.5	77.3
Contralateral side	62	109	7.3	112.2 (± 42.5)	106.3 (± 37.9)	− 5.9 (± 41.1)	− 5.2	49.5

*CBFV* cerebral blood flow velocity, *MCA* middle cerebral artery, *SD* standard deviation

**Fig. 1:**
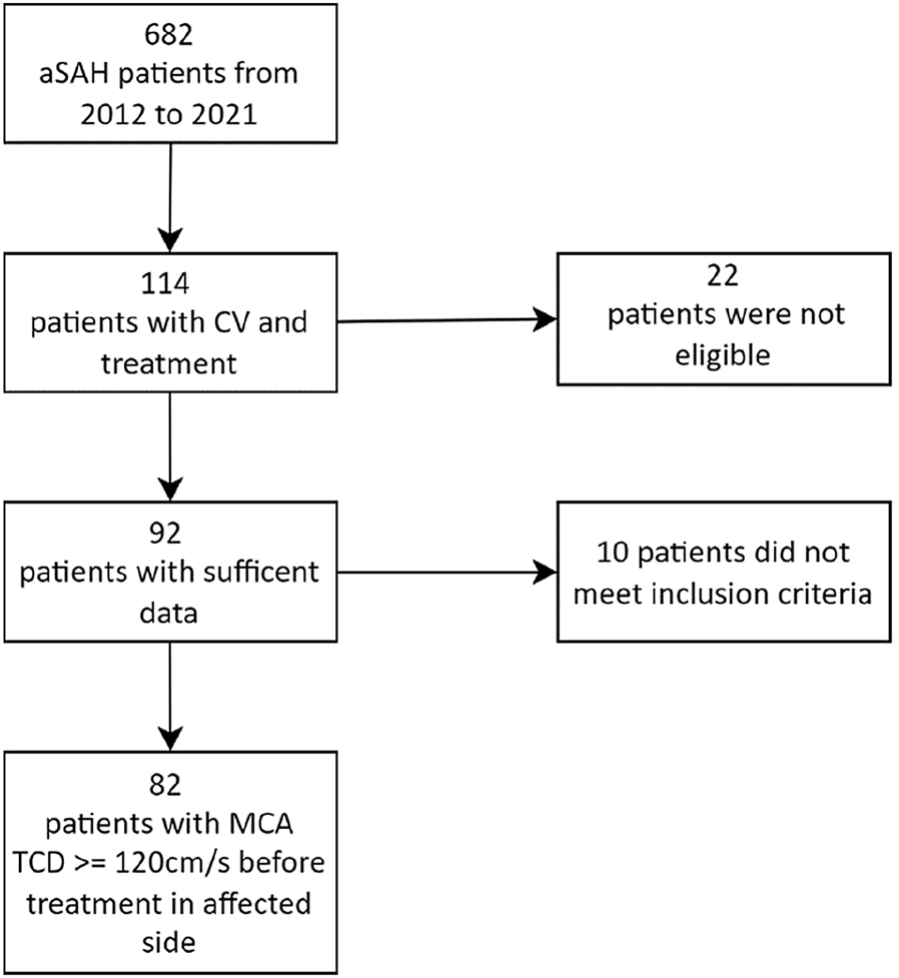
Patient selection from the patient data management system. A total of 22 patients were not eligible because of missing values. Ten patients with IAS treatment and CV with cerebral angiography did not meet the inclusion criteria (preintervention TCD of MCA ≥ 120 cm/s). aSAH, aneurysmal subarachnoid hemorrhage, CV, cerebral vasospasm, IAS, intraarterial spasmolysis, MCA, middle cerebral artery, TCD, transcranial Doppler

Among 109 areas treated with SGB, in 74 (67.9%) the CBFV remained lower after 24 h with respect to the initial value (mean difference: − 30.1 cm/s; standard deviation ± 45.2). After IAS, 58 of 75 areas (77.3%) remained lower after 24 h with respect to the initial CBFV (mean difference: − 31.5 cm/s; standard deviation ± 45.2). In the control group, in 54 of 109 patients (49.5%), the CBFV after 24 h remained lower than the initial value (Fig. 1c).

**Fig. 2:**
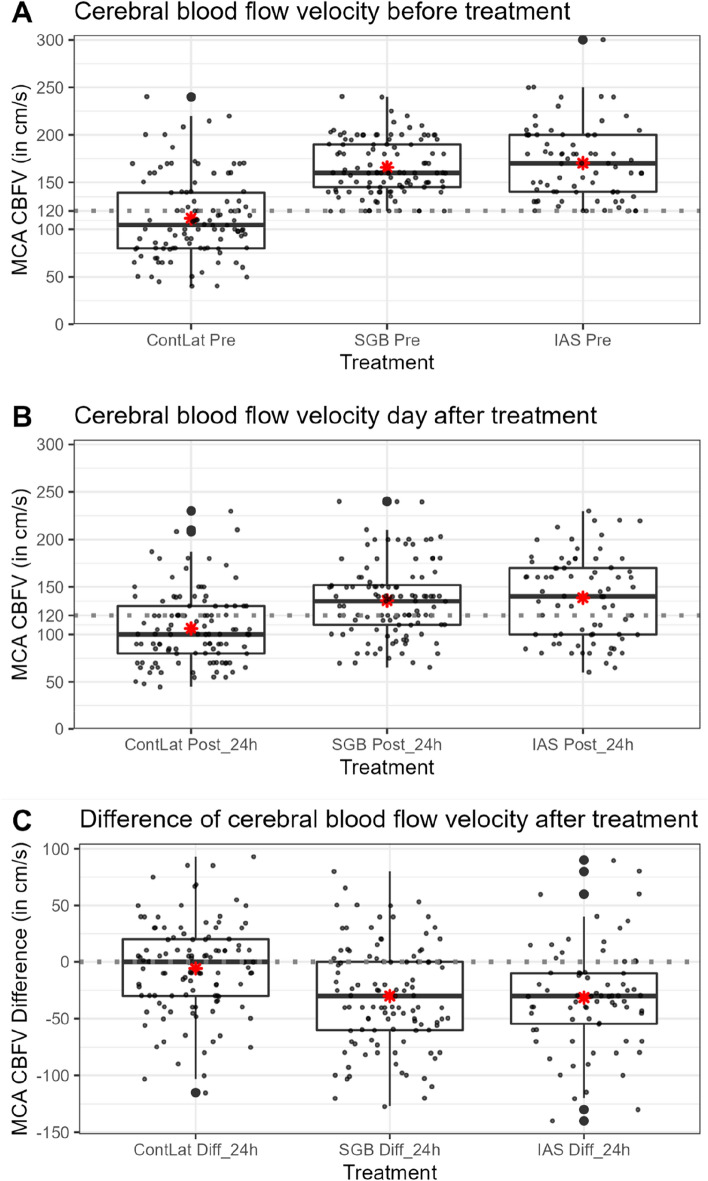
MCA CBFV before treatment (**a**) and on the following day (**b**), and difference (**c**) in treatment groups after 24 h (ContLat, SGB, and IAS). *mean; ContLat *n* = 109, SGB *n* = 109, IAS *n* = 75. CBFV, cerebral blood flow velocity, ContLat, contralateral side, Diff, difference, IAS, intraarterial spasmolysis, MCA, middle cerebral artery, SGB, stellate ganglion block

Differential values pre-TCD and post-TCD showed a normal distribution in histograms across all treatment groups. Eleven of 82 patients (13.4%) received continuous intraarterial nimodipine infusion through microcatheters during the ICU stay as a last-resort therapy (Fig. 2).

The mixed linear model for the difference in the MCA CBFV before treatment and 24 h after treatment showed significantly lower CBFV for both treatments compared with those at the contralateral side with no treatment. However, large confidence intervals were observed for all treatments. Based on the estimates, SGB appeared to lead to a slightly greater decrease of CBFV (− 24.59 cm/s) compared to IAS (− 23.46 cm/s), but with widely overlapping confidence intervals, no treatment demonstrated a significant advantage over the other.

To reveal additional factors relevant to the MCA CBFV, decrease after treatment, we added the additional parameters of sex, age, treatment of aneurysm (clipping/coiling), WFNS, and Fisher score, with a random intercept relating to the participating patient. None of these factors showed a significant effect on CBFV decrease (Table 3, Supplemental Fig. S3).

**Table 3:** Fixed and random effects and CI of the mixed linear model

MCA CBFV difference model			
Fixed effects	Estimates	95% CI	*p* value
Intercept	− 23.33	− 84.44 to 37.69	0.450
SGB	− 24.59	− 35.86 to − 13.32	< 0.001*
IAS	− 23.46	− 36.28 to − 10.65	< 0.001*
Sex (male)	− 1.47	− 15.41 to 12.47	0.834
Age	0.36	− 0.32 to 1.05	0.293
WFNS	1.92	− 3.13 to 6.96	0.450
Fisher	1.04	− 10.11 to 12.19	0.853
Coiling	− 10.12	− 45.65 to 25.41	0.574
Clipping	− 8.92	− 45.21 to 27.37	0.628

*p* values are based on the Kenward–Roger approximation; *p ≤ 0.05 was considered statistically significant. Observations 293 in 82 Participants; Marginal *R*^2^ 0.075; Residual variance 1662.62

*CBFV* cerebral blood flow velocity, *CI* confidence interval, *IAS* intraarterial spasmolysis, *MCA* middle cerebral artery, *SGB* stellate ganglion block, *WFNS* World Federation of Neurological Surgeons

In a subgroup analysis of patients treated with SGB, our mixed linear regression model indicated that the addition of clonidine (*n* = 52/109) did not lengthen effect on CBFV decrease after 24 h, but this effect was not significant (Supplemental Table S2).

Twenty-two patients (22/46, 48%) initially treated with SGB showed no significant response after 2 h in TCD and did not improve neurologically, and subsequent IAS was received in 33 areas (33/75, 44%). Subgroup analysis of this small cohort revealed higher pretherapy CBFV 178.8 (± 40.1) cm/s, as well as higher CBFV 24 h after treatment 142.9 (± 41.6) cm/s, resulting in a higher relative mean MCA decrease (− 20.1%). The decrease persisted in 78.8% of patients after 24 h (Supplemental Table S3).

### Outcomes

In our cohort of patients with CV after aSAH, 34/82 (41%) had cerebral infarction on magnetic resonance imaging or computed tomography after ICU stay, and 50/82 (61%) of patients were discharged with unfavorable GOS (≤ 3). Seven patients died during the ICU stay (9%). A total of 8/82 patients (10%) were lost to follow-up (Supplemental Table S2).

**Fig. 3:**
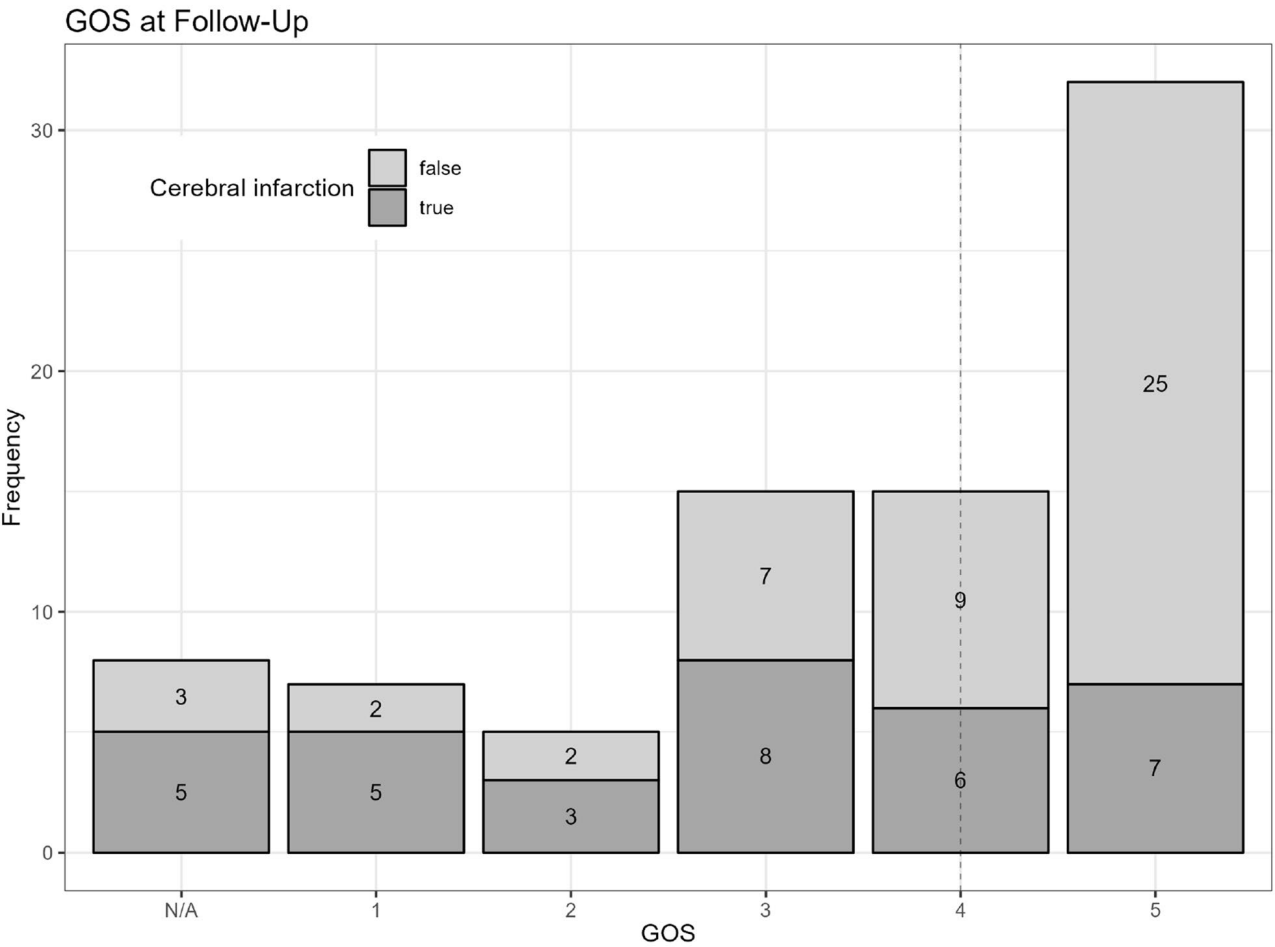
Histogram of GOS at follow-up and the presence of cerebral infarction at ICU discharge in each GOS group (*n* = 82). Dashed line indicates the median GOS at follow-up (*n* = 74). Eight patients were lost to follow-up, of which seven patients had poor outcomes (GOS 2–3) at discharge, and one patient had GOS 4. Seven patients died during the ICU stay (GOS 1). GOS, Glasgow Outcome Scale, ICU, intensive care unit, N/A, not available

A total of 20/67 of survivors (30%) had severe impairment 6 months after the incident (GOS 2–3); in comparison, 47/67 (70%) had excellent clinical outcomes (GOS ≥ 4), including 27 patients with an unfavorable GOS (≤ 3) at discharge (Fig. 3).

Logistic regression for unfavorable GOS at follow-up (GOS ≤ 3), found the presence of cerebral infarction at discharge to be a significant predictor for outcome (Table 4).

**Table 4:** Logistic regression for unfavorable GOS (≤ 3) at follow-up

Unfavorable GOS at follow-up (GOS ≤ 3)			
Parameter	Odds ratios	95% CI	*p* value
Age	1.05	0.99–1.12	0.139
Sex (male)	1.03	0.30–3.51	0.956
Cerebral infarction present	6.37	2.06–22.03	0.002*
WFNS	1.17	0.75–1.89	0.504
Fisher	1.93	0.80–5.70	0.174
Coiling	0.20	0.02–2.08	0.188
Clipping	0.18	0.01–2.15	0.187

*p ≤ 0.05 was considered statistically significant. Observations 72; Akaike information criterion = 94.393; *R*^2^ = 0.239

*CI* confidence interval, *GOS* Glasgow Outcome Scale, *WFNS* World Federation of Neurological Surgeons

No complications were observed after SGB. In the IAS group, four patients experienced a blood pressure decrease while milrinone was applied, thus resulting in a lower dose than the intended 8 mg of milrinone per area.

## Discussion

Data from our mixed linear regression model support the use of SGB as a readily available bedside treatment option for patients with symptomatic CV. This treatment led to a significant and 24 h lasting decrease in MCA CBFV on the affected side. Similarly, IAS with milrinone, a widely used rescue therapy in patients with severe CV, shows a comparable decrease in CBFV in MCA. To reveal potential covariates, our mixed model includes other possible variables (sex, age, WFNS, Fisher score, and aneurysm treatment modality). However, only treatment with SGB and IAS significantly affected the CBFV, but none of the variables. Hence, our findings indicate that both therapies significantly decrease CBFV in treated areas, while contralateral areas remain unaffected.

Patients with high-grade aSAH are often mechanically ventilated and sedated, thus complicating the detection of neurological deficits and DCI in the acute phase. Furthermore, 21% of patients with symptomatic CV, who solely receive hypertensive therapy do not respond to medical treatment. Treatment failure is associated with a risk of death or severe disability after 1 year.

TCD is widely used as a bedside CV screening tool. In comparison with angiography, TCD can predict symptomatic vasospasm with high sensitivity, specificity, and positive predictive values (Supplemental Table S5). A decrease in CBFV correlates inversely with the vessel diameter and suggests an increase in cerebral blood flow. A symptomatic CV cutoff of a mean CBFV of 120 cm/s and higher in MCA is described in the literature as a symptomatic CV [24].

SGB is a regional anesthetic block long known to resolve clinical deterioration in patients with CV. The cervical sympathetic ganglia supply sympathetic fibers to the ipsilateral cerebral vessels. A blockage decreases the sympathetic tone and leads to a decrease in ipsilateral vasoconstriction [25].

Often used rescue therapies for refractory CV include chemical angioplasty (IAS) or balloon dilatation. In refractory CV therapy can be escalated to microcatheters for continuous intraarterial nimodipine application. Balloon angioplasty is believed to have longer-lasting effects but is mechanically limited to the proximal vasculature, whereas IAS is thought to also affect the small vessels. In the literature, IAS leads to an excellent angiographic response: 60% of patients show postprocedure neurological improvement, and 66% show good clinical outcomes (GOS 4–5) at follow-up.

IAS with milrinone, a phosphodiesterase-3 inhibitor, beneficially reverses CV by leading to smooth muscle relaxation in the arterial and venous vasculature. Subsequently, vasodilation leads to increased cardiac output [6, 8, 9].

In our cohort with TCD and angiography for IAS patient selection, we observed a lasting CBFV response (− 31.5 cm/s) on the day after treatment, whereas in the SGB group the response on CBFV was − 30.1 cm/s. Our data showed a lasting effect for at least 24 h on MCA CBFV in 67.9% of patients after SGB and 77.3% after IAS. The SGB data are comparable to our 2019 findings [10].

On the basis of our subgroup analysis, we cannot conclude that addition of clonidine to the SGB has a potential benefit for prolonging block duration (Supplemental Table S2) [10, 13, 14].

Conservative treatment failure is associated with a higher risk of new infarction, the primary determinant of long-term outcomes after aSAH. A total of 62% of patients who failed medical treatment are thought to be dead or severely disabled (GOS 1–3) at 12 months, whereas only 25% of patients who responded to medical treatment had GOS 1–3. Although the outcome benefits of endovascular intervention remain unproven, early implementation of rescue therapies for refractory symptomatic vasospasm may improve outcomes in these patients. In 2021, Zhang et al. [16] implemented SGB on days 1, 3, and 7 after clipping following aSAH and showed a better outcome for the SGB group compared with the non-SGB group.

In our cohort of patients with refractory CV, good clinical outcomes were seen in 70% of surviving patients (47/67) at follow-up (GOS ≥ 4). Nevertheless, 30% of the surviving patients remained disabled (20/67) at follow-up. During the ICU stay, 7 of 82 patients died, and 8 of 82 were lost to follow-up (Supplemental Table S4). Thus, we assume that their clinical condition had not substantially changed [6].

A study in 2021 reported that 68% of patients (86/126) with DCI following aSAH showed new infarctions on imaging. In DCI group 67% had an unfavorable neurological outcome at discharge (84/126), compared with 43% (92/219) in the non-DCI group.

In the present study, 61% of patients had unfavorable neurological outcomes at discharge (50/82), whereas cerebral infarction from DCI was identified in 41% of patients (criteria in Supplemental Methods 1 and data in Supplemental Table S4). Our DCI cohort shows a significantly lower incidence in cerebral infarctions (41% vs. 68%), but unfavorable neurological outcomes at discharge are comparable (61% vs. 67%) [26].

In 2021, a study described the combination of SGB and IAS as a “one-stop shop” for CV. For this study, 19 SGB procedures were performed in ten patients with CV after the failure of traditional hemodynamic and endovascular treatments. Adjuvant SGB combined with intraarterial therapy within a single session was believed to be beneficial. The effect on the distal vasculature was thought to be like that of intraarterial nimodipine administered through a microcatheter. This supports the findings of our small subgroup with patients treated with SGB who received subsequent IAS (combined treatment). The combined group shows a good response of CBFV after 24 h (− 20.1%) and a lasting decrease in 78.8% of patients, compared with 67.9% in SGB group. The combined group exhibited a 7.3% higher initial CBFV (combined 178.8 vs. 165.8 cm/s SGB group; Supplemental Table S3) [27].

No major complications resulting from SGB or IAS were observed during our study period. However, possible risks from SGBs include bleeding, puncture of the carotid artery with subsequent dissection or thrombotic occlusion, infection at the injection site, and allergies.

IAS with milrinone can lead to severe hypotension, arrhythmia, hyperkalemia, hypomagnesemia, myocardial ischemia, and common procedure-associated risks, such as artery dissection or bleeding.

### Strengths and Limitations

This is the most extensive study, to date, comparing CBFV in patients with CV after SGB and IAS using the untreated contralateral side as a control. The study population is consistent with people typically at risk of DCI after high-grade aSAH.

The main study limitation is its retrospective nature, as all study parameters were extracted from the available ICU data. A distinct difference in sample size existed between treatment groups.

Nonresponders to SGB after 2 h were subsequently treated with IAS, which lead to 22 patients overlapping in treatment groups.

Regarding our statistical analysis, all treatments appeared strong in the mixed linear model, but the confidence intervals were broad and showed overlap. Therefore, we cannot conclude that one therapy is superior to another.

TCD ultrasonography is widely accepted as a first-line bedside tool in neurocritical care practice for assessing CBFV. However, its high sensitivity depends on user experience and the quality of the patient's acoustic bone window.

Beyond neurological examination and TCD after 2 h, no alternative control for the effectiveness of SGB was performed. Horner syndrome, unilateral sweating, or other clinical signs of SGB effectiveness were not noted.

## Conclusions

Our study shows promising results suggesting a lasting decrease in CBFV after SGB and IAS in patients with CV. Early SGB could be a beneficial bedside treatment for CV.

We suggest SGB for patients with mild symptomatic CV (CBFV < 180 cm/s), and subsequent escalation to IAS proved to be helpful in patients with severe CBFV elevation (CBFV ≥ 180 cm/s). Randomized controlled trials on (single) therapy strategies, focused on patient outcomes and incorporating vasopressor dependence are needed for more precise evaluation (i.e., BLOCK-CVS) [28].

### Supplementary Information

Below is the link to the electronic supplementary material.

Supplementary file1 (DOCX 321 kb)
