# Supplementary material for: Stellate Ganglion Block and Intraarterial Spasmolysis in Patients with Cerebral Vasospasm: A Retrospective Cohort Study
Source: Neurocrit Care. 2023 Jul 27;40(2):603–11. doi: 10.1007/s12028-023-01762-w (PMC10959776; doi:10.1007/s12028-023-01762-w)
Supplement: Supplementary file 1 — Supplementary file1 (DOCX 321 kb) [file 12028_2023_1762_MOESM1_ESM.docx]

# Supplemental materials

### Supplemental methods

### Radiologic criteria for infarction

Radiologic criteria for cerebral infarction relating to delayed cerebral ischemia (DCI), according to a 2010 consensus^17^:

Presence of cerebral infarction on CT or MR scan within 6 weeks after SAH or on the latest scan made before death within 6 weeks and:

- - not present on CT or MR scan 24–48 hours after aneurysm closure
  - not attributable to endovascular or surgical procedures

### Criteria for study inclusion

| **Inclusion criteria:** | Aneurysmal subarachnoid hemorrhage |
| --- | --- |
|  | MCA CBFV on affected side ≥120 cm/s |
|  | Sudden onset of neurological deficit, or Glasgow coma scale (GCS) decrease,  or intubated and mechanically ventilated |
|  | Rescue treatment for CV (IAS or SGB) |
|  | All values available (except for GOS on follow-up) |
|  |  |

### Supplemental tables

### Descriptive data for included therapies

|  |  | SGB | IAS | ContraLateral |
| --- | --- | --- | --- | --- |
| Count | **N (F/M)** | 60 (36/24) | 46 (32/14) | 62 (39/23) |
|  | **Aneurysm location (AC/PC/none)** | 52/8/0 | 39/6/1 | 53/9/0 |
|  | **Coiling/clipping/none** | 35/22/3 | 28/16/2 | 37/23/2 |
|  | **Cerebral Infarction** | 26 | 21 | 28 |
| Mean  (± sd) | **Age (years)** | 49.7  (± 10.8) | 49.1  (± 9.6) | 49.6  (± 9.5) |
|  | **Days to aneurysm treatment** | 0.9  (± 1.6) | 0.9  (± 1.6) | 1.1  (± 1.9) |
|  | **ICU LOS (days)** | 23.7  (± 10.0) | 25.2  (± 9.0) | 22.8  (± 9.6) |
| Median  [IQR] | **WFNS scale** | 4 [2–5] | 4 [3–5] | 4 [2–5] |
|  | **Fisher scale** | 4 [4–4] | 4 [4–4] | 4 [3–4] |
|  | **GOS at discharge** | 3 [2–4] | 3 [2–4] | 3 [2–4] |
|  | **GOS at follow up** (N=74) | 4 [3–5] | 4 [3–5] | 4 [3–5] |

Table S1: Descriptive data of study population sorted in different therapy regimens. SGB: Stellate ganglion block, IAS: Intra-arterial spasmolysis, N: Number, F: Female, M: Men, AC: Anterior circulation, PC: Posterior circulation, sd: standard deviation, IQR: Interquartile range, LOS: Length of stay, WFNS World Federation of neurological surgeons, GOS: Glasgow Outcome Scale.

### Effect of clonidine addition in the SGB subgroup

|  | Clonidine’s influence on  MCA difference after 24 h | | |
| --- | --- | --- | --- |
| Fixed effects | **Estimates** | **CI (95%)** | **p** |
| (Intercept) | -34.02 | -46.35; -21.69 | **<0.001** |
| Clonidine true | 8.30 | -9.53; 26.13 | 0.357 |
|  |  |  |  |
| Random effects | | | |
| σ² | 2009.38 | | |
| τ00 ID | 36.01 | | |
| N ID | 60 | | |
| Observations | 109 | | |
| Marginal R^2^ / conditional R^2^ | 0.008 / 0.026 | | |

Table S2: Mixed linear regression model for decrease in CBFV after SGB with clonidine addition, with a random intercept relating to the participating patient, fitted with restricted maximum likelihood. p ≤ 0.05 was considered statistically significant. CI: confidence interval, σ²: Residual variance, τ00: Random intercept variance, N ID: Number of patients included.

### Added subgroup analysis of combined treatment group and IAS alone

|  | **Count** | |  | **CBFV in cm/s** | | | **CBFV in percentage** | |
| --- | --- | --- | --- | --- | --- | --- | --- | --- |
|  | **Patients** | **Areas** | **Mean day of treatment** | **Mean MCA pre** | **Mean MCA after 24 h** | **Mean MCA difference 24 h** | **Mean MCA decrease** | **Decrease persistent after 24 h** |
| **Stellate ganglion block** | 60 | 109 | 7.3 | 165.8  (± 29.3) | 135.7  (± 39.5) | -30.1  (± 45.2) | -18.2% | 67.9% |
| **Intra-arterial spasmolysis** | 30 | 42 | 8.6 | 163.4  (± 37.8) | 135.3  (± 46.7) | -28.1  (± 44.1) | -17.2% | 76.2% |
| **Combined (SGB + IAS)** | 22 | 33 | 7.9 | 178.8  (± 40.1) | 142.9  (± 41.6) | -35.9  (± 46.8) | -20.1% | 78.8% |
| **Contralateral side** | 62 | 109 | 7.3 | 112.2  (± 42.5) | 106.3  (± 37.9) | -5.9  (± 41.1) | -5.2% | 49.5% |

Table S3: Means and standard deviations (± sd) of the CBFV decrease in each group.

### GOS at discharge

|  | GOS  1 | GOS 2–3 | GOS 4–5 | Lost | Sum |
| --- | --- | --- | --- | --- | --- |
| Discharge | 7 | 43 | 32 | - | 82 |
| - Infarction | 5 | 22 | 7 | - | 34 |
| Follow Up | 7 | 20 | 47 | 8 | 82 |
| - Infarction | 5 | 11 | 13 | 5 | 34 |

Table S4: GOS at discharge and follow up (at 6–12 months) compared to count of cerebral infarctions at discharge. GOS: Glasgow Outcome Scale

### DCI prediction of TCD and cerebral angiography

| **Prediction of DCI** | **Cerebral angiography (%)** | **TCD (%)** |
| --- | --- | --- |
| Sensitivity | 57 | 90 |
| Specificity | 68 | 71 |
| Positive predictive value | 32 | 57 |
| Negative predictive value | 90 | 92 |

Table S5: Comparison of angiography vs TCD for DCI ^4,19^

### Supplemental figures

###
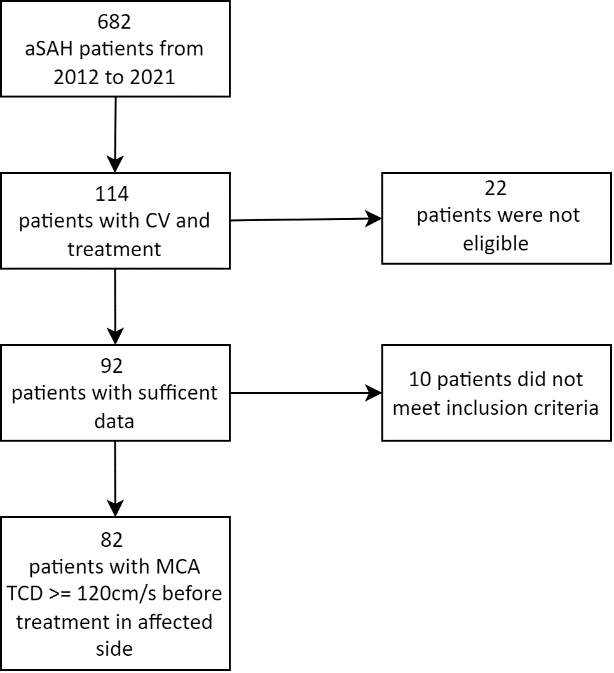
Flowchart of patient selection

Figure S1: Patient selection from the patient data management system. A total of 22 patients were not eligible because of missing values. Ten patients with IAS treatment and CV with cerebral angiography did not meet the inclusion criteria (preintervention TCD of MCA ≥120 cm/s).

### Therapy strategy Flowchart


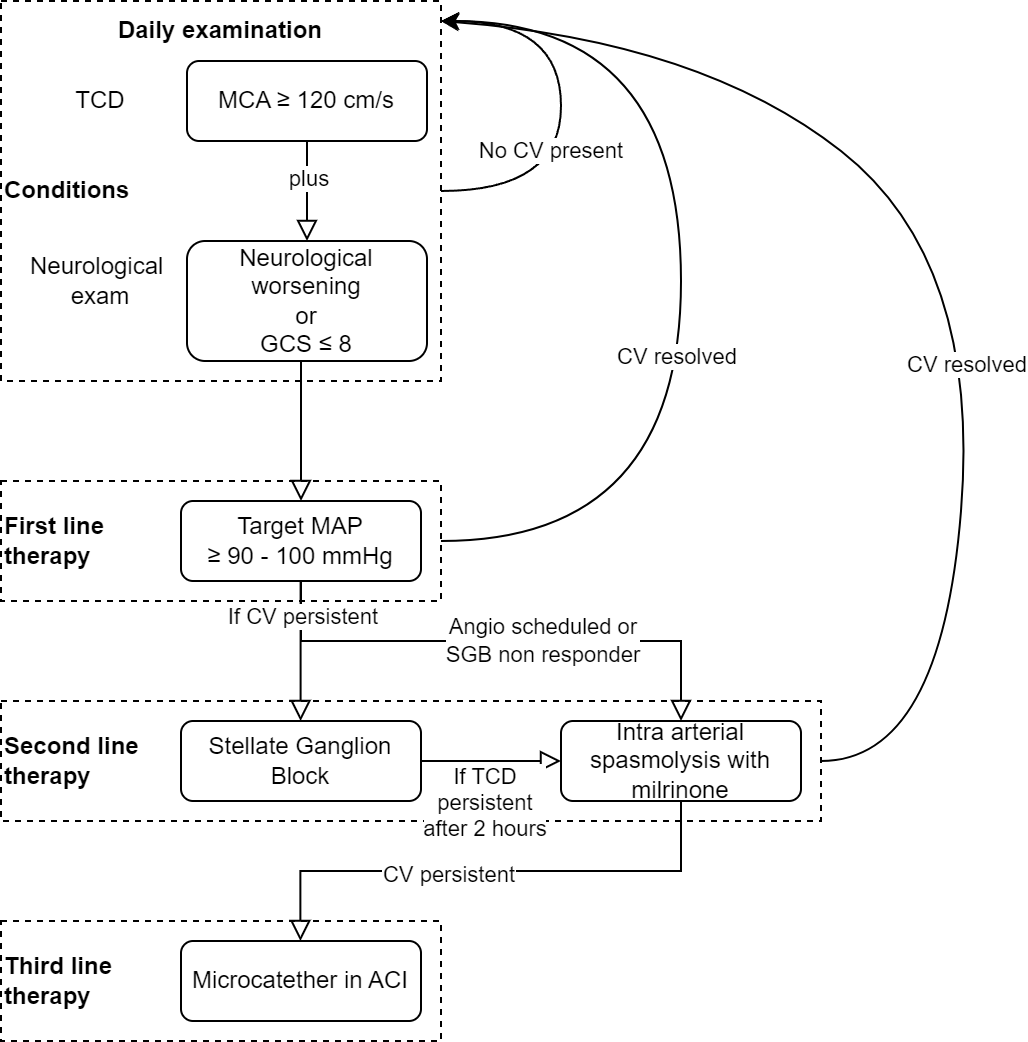


Figure S2: Selection and escalation of therapies after detection of clinical symptomatic CV. TCD: Transcranial doppler, MCA: Middle cerebral artery, CV: Cerebral vasospasm, GCS: Glasgow coma scale, MAP: Mean arterial pressure, SGB: Stellate ganglion block, ACI: Arteria carotis interna

### Dot and whisker plot of fixed effects on MCA difference


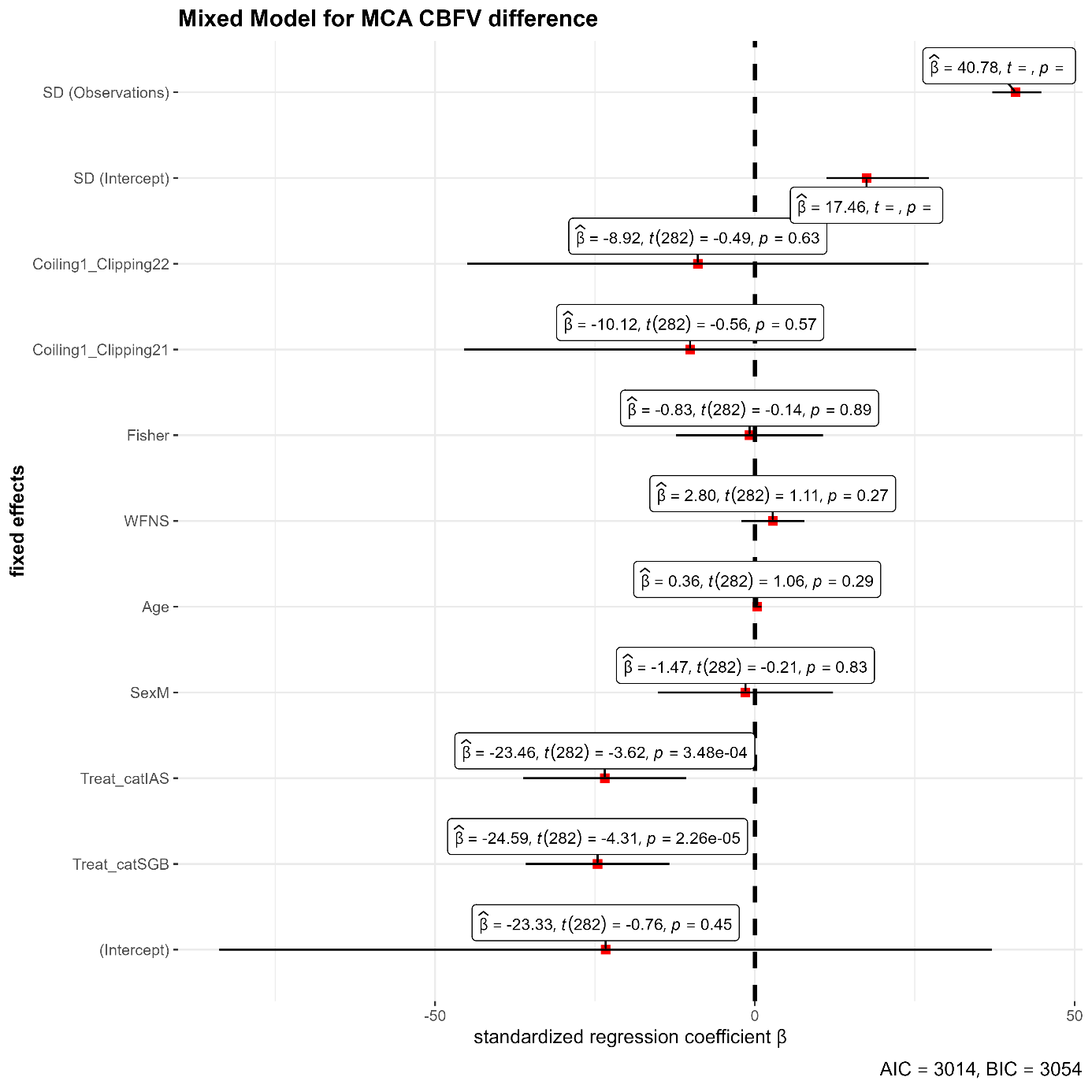


Figure S3: Dot and whisker plot for mixed model linear regression on MCA difference the day after treatment (Table 3). (Created with Patil, I. (2021). Visualizations with statistical details: The 'ggstatsplot' approach. Journal of Open Source Software, 6(61), 3167, doi:10.21105/joss.03167.)
